# Physicochemical and Nutritional Characteristics of Cookies Prepared with Untapped Seaweed *Ulva intestinalis*: An Approach to Value Addition as a Functional Food

**DOI:** 10.3390/foods12010205

**Published:** 2023-01-03

**Authors:** Md. Mohibbullah, Al Amin, Md. Abu Talha, Md. Abdul Baten, Md. Masud Rana, Ashfak Ahmed Sabuz, Asif Wares Newaz, Jae-Suk Choi

**Affiliations:** 1 Department of Fishing and Post Harvest Technology, Sher-e-Bangla Agricultural University, Dhaka 1207, Bangladesh; 2 Postharvest Technology Division, Bangladesh Agricultural Research Institute, Gazipur 1701, Bangladesh; 3 Department of Seafood Science and Technology, The Institute of Marine Industry, Gyeongsang National University, 38 Cheondaegukchi-gil, Tongyeong-si 53064, Republic of Korea

**Keywords:** green algae, *Ulva intestinalis*, cookies, sensory evaluation, physicochemical and nutritional properties, functional food

## Abstract

The present study was investigated to know the sensory, physicochemical, nutritional and fatty acid properties of seaweed-based cookies prepared with untapped seaweed *Ulva intestinalis* (UI) from Bangladesh coast. The cookies were formulated with different percentages of UI inclusions both in powdered (PUI) and fragmented (FUI) forms, in order to evaluate different quality attributes in prepared value-added cookies. In sensory analysis, seaweed inclusion levels of 1% PUI, 2.5% PUI, 1% FUI, 2.5% FUI and 5% FUI to cookies were acceptable by panelists. Considering the maximum percentage of seaweed inclusions, 2.5% PUI and 5% FUI were selected for further analysis. The results of physicochemical properties such as moisture content, spread factor, baking loss, pH, cookie density, color, texture properties, volatile basic nitrogen and thiobarbituric acid reactive species were within acceptable limits. In nutritional analysis, 2.5% PUI and 5% FUI cookies showed a remarkable and significant increase in lipid and ash contents, compared to untreated controls. Being the first report on fatty acids profile by UI from Bangladesh, among 24 fatty acids identified, the amount of total saturated, mono-unsaturated, omega-3 fatty acids and omega-6 fatty acids were reported to be 641.9 (36.2%), 563.7 (31.8%), 133.8 (7.6%) and 436.3 (24.6%) μg/g DW, respectively. The results suggest that cookies with 2.5% PUI and 5% FUI can be marketed as healthy foods for consumers.

## 1. Introduction

Seaweed refers to thousands of species of macroscopic, multicellular, marine algae. Seaweeds adhere to rocks in the intertidal zone, wash up on the beach, and float on the sea surface. Seaweeds are classified taxonomically into the following three groups: Chlorophyta, Rhodophyta, and Phaeophyta, corresponding to green, red and brown algae, respectively. More than 20,000 seaweeds are distributed around the world of which only 221 (1.1%) are commercially exploited, including 145 species for foods, including raw salad, curry, soup, pickles, cookies, etc., and 110 species for phycocolloids, including agar, agarose, carrageenan, algin, and mannitol [1,2]. Moreover, seaweeds have the potential to be used as medicine, cosmetics, animal feed, fish feed, fertilizers, soil conditioners, etc. [3]. A recent study also confirms that the use of sea grapes (*Caulerpa racemosa*) in cookies is a potential anti-aging novel-functional food [4].

In Bangladesh, on the south coast bordering the Bay of Bengal, from the Sundarban mangrove forest to the island of St. Martin, seaweeds are available from October to April [5]. More than 77 genera and approximately 250 species of seaweeds are found in the coastal and near-coastal waters of Bangladesh [6]. In Bangladesh, seaweed farming has been practiced in the tidal and shallow subtidal area characterized by a rocky coral substrate, sandy bottom with boulders, pebbles and broken shells as well as a sandy-muddy bottom that provides a suitable substrate and suitable habitats for the cultivation of various seaweeds.

*Ulva intestinalis* is a conspicuous bright grass-green seaweed, consisting of inflated irregularly constricted, tubular fronds that grow from a small discoid base. Fronds are usually unbranched and can be 10–30 cm or more in length and 6–18 mm in diameter, with the tips usually rounded [7]. *Ulva intestinalis* is remarkably euryhaline as it can grow in freshwater. However, there is evidence for the existence of genetic strains adapted to high and low salinity [8]. It can be found in a variety of habitats on all coastal plains. Given the right conditions, it can grow on rocks, mud, and even sand. This species is commonly found in areas where brackish water is most abundant and is also known as a common epiphyte found on other seaweeds. Availability of this species is reported from the coastal areas of Cox’s Bazar and St. Martins Island, Bangladesh from October to April.

*Ulva intestinalis* is also of great commercial importance. Coastal people take this species as fodder and it is consumed only by the tribal community. A recent study reported that the proximate composition of *U. intestinalis* on a dry basis was crude protein (12.6%), carbohydrate (45.4%), crude fat (3.5%), crude fiber (17.1%) and Ash (12.7%) [9]. However, few studies have been found on the use of *Ulva intestinalis* as a feed ingredient and growth promoter [10,11]. Nowadays, seaweed has become a very versatile product and is widely used as a food for human consumption. Therefore, seaweed can be used as a functional food because it is characterized by low energy content, good sources of polysaccharides, high protein content, good supply of dietary fiber, high tocopherol, high fatty acids, and a rich source of distinguished minerals [12]. For the first time in Bangladesh, value-added seaweed products, such as seaweed-based cookies may be a new approach to market these untapped resources into valuable foods. Because of their unique taste and extended shelf life even at room temperature with appropriate packaging, cookies are considered popular foods regardless of countries in the world. However, the lack of valuable micronutrients and fiber in cookies due to the use of refined grains is of great concern to the health-conscious consumer. Therefore, the present study aimed to develop and evaluate the quality of seaweed inclusion in cookies with its two forms, including powdered *Ulva intestinalis* (PUI) and fragmented *Ulva intestinalis* (FUI), considering physicochemical and nutritional attributes in prepared value-added cookies.

## 2. Materials and Methods

### 2.1. Collection, Identification and Transportation of Seaweed

*Ulva intestinalis* was collected in March 2021 from the coast and tidal area of Nuniachara beach, Cox’s Bazar (Latitude 21°28′27.79″ N and Longitude 91°58′23.00″ E). The seaweed was authenticated by a researcher at the Bangladesh Fisheries Research Institute (BFRI) who was implementing a project to develop and propagate the seaweed culture, and deposited as a voucher specimen of UI-NC:MM-2021-03 in the department of Fishing and Post Harvest Technology, Sher-e-Bangla Agricultural University, Bangladesh. Immediately after harvesting, the sample was placed in a large plastic container with sufficient seawater and transported to the laboratory for further processing.

### 2.2. Processing of Seaweed

Raw seaweed samples were properly washed with running water to remove sand and other debris from *Ulva intestinalis*. The seaweed was dried at room temperature (20–25 °C) for three to five days to reduce the moisture content. Then, dried seaweed was packed in plastic zipper bags and brought to a dark state. The dried seaweed were ground into powder in a mill (Mozart Mixer Grinder, Miyako, Dhaka, Bangladesh) with particle size of less than 50 µm in diameter and, on the other type, were made by hand-pressing into fragmented forms with uneven sizes from 500 µm to 3000 µm. Both samples were packed in airtight plastic zipper bags and stored at room temperature under dark condition for further use.

### 2.3. Preparation of Seaweed Cookie

The basic formulation for making seaweed-based cookies is outlined in Table 1. Seaweed powder has been substituted for wheat flour in varying amounts to fortify the nutritional content of cookies. In this study, 0 (control), 1, 2.5, 5 and 10% of the wheat flour were replaced with seaweeds and these compositions were selected based on previous reports of similar seaweed-based cookies [13,14]. All ingredients including wheat flour, seaweed powder, powdered sugar and butter were accurately weighed using an electric balance (FSH, A&D Company Ltd., Republic of Korea). Dry ingredients such as whole wheat flour (13% protein), sugar powder and seaweed powder were mixed together at room temperature. The butter was softened and then mixed with dry ingredients. The cookie dough was then rolled out with a rolling pin and cut to 5 mm thickness and 51 mm diameter with a cutter and baked in an oven at 180 °C for 12 min. The cookie was vacuum packed in high density polyethylene (HDPE) bags with a seal and placed in airtight containers at room temperature for further analysis.

### 2.4. Sensory Evaluation of Cookies

The cookies containing 1, 2.5, 5 and 10% seaweed powder were subjected to a sensory evaluation along with the control biscuit (0%). This sensory test was conducted by 21 panelists, age between 25 and 40, belonging to the Faculty of Fisheries, Aquaculture and Marine Sciences, Sher-e-Bangla Agricultural University, Dhaka. The panelists were semi-trained and capable of discriminating the differences and communicating their sensory reactions as perceived by the sense of sight, taste and touch. The 10 g biscuit samples from each group were given to the panelists and asked to rate on a 9-point hedonic scale for the attributes of seaweed biscuit appearance, color, odor, texture, taste and overall acceptability. A hedonic scale of 1 to 9 points indicated differently as 1 = dislike extremely, 2 = dislike very much, 3 = dislike moderately, 4 = dislike slightly, 5 = neither like nor dislike (threshold point), 6 = like slightly, 7 = like moderately, 8 = like very much, and 9 = like extremely [15]. The samples were served on white dishes with mineral water, a stainless spoon, a paper cup, plain bread and pre-defined sensory evaluation paper with number rankings. Each sample group was evaluated at different times with the same panelists. All samples were encoded before serving and evaluated randomly. The sensorial properties were evaluated at 3 p.m. for a maximum of 2 h in a designated room. On the day of sensory evaluation, all cookies were freshly prepared at 10 a.m., cooled down at RT after baking, and vacuum-packed until sensory evaluation on the same day at 3 p.m.

### 2.5. Quality Assessment of Dough

The baking sample (5 g) was mixed in 45 mL of distilled water, homogenized for 2 min, and centrifuged (SHG-15D, SciLab, and Seoul, Republic of Korea) to collect the supernatant. The pH of the sample was measured using a pH meter (OHAUS STARTER 2100, Seoul, Republic of Korea). Dough or cookie density (g/cm^3^) was measured by dividing the dough or cookie weight by a piece of dough or cookie used to form a biscuit as reported by Hadi Nezhad and Butler [16]. Moisture content was determined using the standard AOAC method [17]. The baking loss (%) was calculated by a comparison between the biscuit weight and the dough weight. The spread factor of the cookies was determined by dividing the diameter (mm) of a biscuit by its thickness (mm) according to the standard method of AACC [18].

### 2.6. Texture Analysis of Cookie

The texture of seaweed-based cookie was evaluated using the TA.XTPlus Texture Analyzer (Stable Micro Systems, Godalming, UK) equipped with Texture Exponent Lite software version 6.1.14.0 (Stable Micro System, Godalming, UK). During compression and extrusion with 12.5 mm diameter of a cylindrical probe at a rate of 1 mms^−1^, number of attributes were measured including hardness (kg), adhesiveness (mJ), cohesiveness, springiness (mm) and gumminess (g), as described previously [19,20].

### 2.7. Color Analysis of Cookie

The color of the seaweed-based biscuit was evaluated with the Konica Minolta (Tokyo, Japan) CM-700d instrument. The surface color of each sample was calculated from previous reports [19,21,22]

### 2.8. Proximate Composition Analysis

Protein, lipid, carbohydrate, fiber, moisture and ash contents of seaweed cookies were determined using the standard AOAC method [17].

### 2.9. Total Volatile Basic Nitrogen (VBN) Analysis

The VBN value of the cookie was determined by a Conway microdiffusion method, as described by Mohibbullah et al. [19]. Briefly, 5 g of a ground sample was homogeneously mixed with 25 mL of distilled water. The filtrate was then mixed with potassium carbonate and incubated at 37 °C for 90 min. After titration with 0.01 N sodium hydroxide, the VBN value was calculated by the following equation:

(1) VBN (mg%)=0.14 × [(b−a) × f/W] × 100 × d

where *b* = the amount of NaOH needed to titer Blank, *a* = the amount of NaOH needed to titer sample, f = 1.003 (NaOH), W = weight of the sample, d = dilution factor

### 2.10. Thiobarbituric Acid Reactive Substance (TBARS)

For the determination of TBARS, a total of 5 g of the ground sample was thoroughly mixed with 20% trichloroacetic acid solution, homogenized for 1 min and filtered using a Whatman No. 1 filter paper (55 mm), as described previously [23]. The filtrate was mixed with 0.005 M thiobarbituric acid solution, incubated at 95 °C for 30 min, and measured absorbance value at a wavelength of 530 mm. The TBARS was calculated by the following equation:

TBARS (MDA mg/1000 g) = [Sample Absorbance − Blank (Water) Absorbance] × 5.2(2)

### 2.11. Soxhlet Extraction

Oil was obtained from finely ground seaweed. A 5 g sample was taken in a thimble and placed in a Soxhlet extractor. The extraction was carried out with diethyl ether (purity 99%) and heating at 45 °C for 5 h. Thereafter, the solvent was removed with a rotary evaporator at 40 °C and then with a vacuum nitrogen dryer until completely dry. The extracted yield of UI oil was 0.96% (*w*/*w*).

### 2.12. Fatty Ccid Analysis

Gas chromatography (Shimadzu Corp., Kyoto, Japan) equipped with a flame ionization detector (GC-FID) was used to identify and quantify the fatty acids from the seaweed sample. Fatty acid methylation of previously extracted oil (0.05 g) was performed according to the American Oil Chemists Society procedure, as followed by the addition of 3 mL 0.5 N NaOH, 3 mL boron trifluoride complex and 10% sodium chloride [24]. Fatty acid methyl esters (FAMEs) were filtered prior to GC injection. An Agilent HP-88 column (100 m, 0.25 mm, 0.2 m) with an oven temperature of 250 °C started from 120 °C, 1 min, 10 °C/min to 175 °C, 10 min, 5 °C/min to 210 °C, 5 min, 5 °C/min to 230 °C, 25 min, 5 °C/min. Helium with a split ratio of 1:20 was used as carrier gas. The FAMEs of the seaweed sample were identified and quantified by comparing standard fatty acids. FAMEs mixture of 37 components of the standard of C4–C24 saturated and unsaturated fatty acids (Sigma-Aldrich Inc., St. Louis, California, USA) were injected into GC/FID to identify the different fatty acids in methyl esterified oil sample extracted from seaweed, operated by windows 7-based software system (GC solution, version 2, Shimadzu Co., Kyoto, Japan). The average fatty acids content was taken from three different chromatograms of individual sample injections.

### 2.13. Statistical Analysis

The SPSS (statistical package for social sciences) 16 (SPSS 2010) statistical package was used for the analysis of the experimental results. The homogeneity of variance was examined using the Levene test. When Levene’s test did not indicate significant deviations from the homogeneity of variance, the data obtained were analyzed by a one-way ANOVA test followed by Tukey and Duncan’s multiple range tests to determine which pairs of the group comparison were significantly different (*p* < 0.05). Results were expressed as mean ± standard error (SE).

## 3. Results and Discussion

### 3.1. Comparison of Different Seaweed Percentage in Biscuit Preparation by Sensory Characteristics

**Figure 1:**
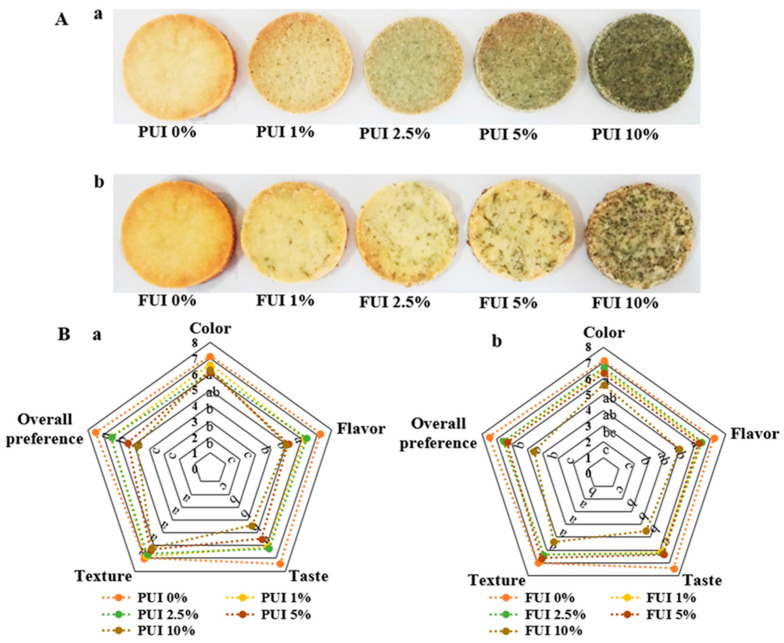
Comparison of different seaweed percentages in biscuit preparation for sensory characteristics. (**A**) Preparation of cookies using powdered UI (**a**) and fragmented UI (**b**). (**B**) Organoleptic analysis of cookies prepared using different concentrations of powdered (**a**) and fragmented (**b**) UI using a radar chart. Data represent the mean ± SE of 3 observations, where groups not sharing a letter are expressed as significantly different (*p* < 0.05).

Cookies of powdered and fragmented UI at different concentrations were shown in Figure 1A. One of the easiest and simplest ways to evaluate consumer products is sensory evaluation, which is associated with various sensorial parameters, such as color, odor, taste, and overall preference; scores for each attribute are based on a 9-point hedonic scale [24]. In the case of the radar chart (Figure 1Ba), the scores of PUI 1% and PUI 2.5% were found almost similar and close to PUI 0% (control), and the values were found on a hedonic scale greater than 6, respectively, which indicated ‘like slightly’ on the quality chart [14], whereas PUI 0% had an average mean value of sensory scores equal to or slightly greater than 7, which indicated ‘like moderately’. In contrast, PUI 5% and PUI 10% showed significantly (*p* < 0.05) lower sensory scores compared to PUI 0%, PUI 1% and PUI 2.5%. Results indicated that there were a significant drop in taste, flavor and overall performance scores when more than 2.5% PUI was added, it might be due to the cause of the increasing fishy odor from seaweed materials. Due to the fact that, the scores remained equal or less than 5 on a hedonic scale, which indicated ‘neither like nor dislike’ or ‘dislike slightly’, respectively [15]. Moreover, in Figure 1Bb, FUI 1%, FUI 2.5%, and FUI 5% treated cookies showed similar sensory scores of greater than 6, which indicated ‘like slightly’ on the quality chart [15], whereas, PUI 0% showed slightly greater than 7 scores, which indicated ‘like moderately’ but a non-significant difference with FUI 1%, FUI 2.5% and FUI 5% groups. However, FUI 10% showed a significantly lower (*p* < 0.05) value compared to FUI 0%, FUI 1%, FUI 2.5%, and FUI 5%, respectively. The value was found an equal or less than 5 on a hedonic scale, which indicated ‘neither like nor dislike’ or ‘dislike slightly’, respectively [15]. The value we found for the sensory evaluation in our present study was higher than that of Oh et al. [13], where the author gave an average score of 5, which is due to the fishy odor of seaweed in prepared cookies. However, in our study, we were able to successfully suppress the dominance of the fishy smell in cookies with the inclusion of a high percentage of UI during preparation. Based on the sensory evaluation, we selected 2.5% PUI and 5% FUI seaweed for the preparation of value-added cookies. Hence, it is indicated that those value-added cookies can be accepted by the consumer and used as a functional food in the market.

### 3.2. Quality Characteristics of Dough Prepared with PUI and FUI

Table 2 examined the quality characteristics of dough prepared with UI seaweed. The pH value is used to indicate the food product quality during processing, as such product appearance, aroma, texture and taste might be affected with changes of pH values [19]. This study found significantly higher (*p* < 0.05) dough pH in control cookies than in PUI 2.5% and FUI 5% cookies. This could be due to the presence of polysaccharides and phenolic compounds containing carboxyl and sulfate groups in the cookies made from UI. Moreover, the ranges of pH among all groups were 6.51 to 6.59, indicating its acceptability for consumption. This study is evident in the findings of Oh et al. [13], where the author reported that the pH of the dough ranged from 6.96 to 7.18, when various seaweeds were added.

The density of the cookie dough is considered an indicator of the air trapped in the dough during mixing. Compared to the control cookies, a non-significant difference in dough and cookie (after baking) density was found in PUI 2.5% and FUI 5%, respectively, indicating no significant changes of cookie diameter among groups. Although dough density was not affected by the addition of *Hizikia fusiforme* up to 5% in cookies [13,25]. Moreover, the average dough density among all groups was 1.32 and, after baking, they were decreased to 0.94. It was due to the difference in weight loss between cookie dough and cookie after baking.

Moisture content is an important attribute for determining the quality of bakery products. Moisture content and water activity are recognized as key factors that directly influence the hardness of dry foods including cookies [26]. The moisture content of the cookies varied from 3.02 to 3.57% and the moisture content of FUI 5% was significantly lower (*p* < 0.05) than that of PUI 2.5% and control cookies, respectively. The current finding is in the range of Kabirullah et al. [27] analyzed the cookies and found a moisture content ranging from 4.06–4.97%.

Baking loss is the removal of moisture, which influences the texture and staling properties of baked products. No significant difference was observed in baking loss and the values were quite similar, ranging from 7.84 to 8.22 for all groups. This result agrees with the findings of Okpala et al. [28], where the author reported that the baking loss of different biscuit compositions varied between 7.14 and 10.39%. It has been reported that moisture content and baking loss are closely related to the moisture retention capacity of cookies [29]. According to Kotoki and Deka [30], baking loss was influenced by the dough ingredients present in cookies; although, there was not a significant difference in baking loss for either regular cookies or cookies with seaweed inclusions. Values for thickness and diameter of the cookies were found to range from 6.6 to 6.8 and 51 to 51.2, respectively, with no significant difference between groups. The spreading factor was found highest in the control, while the lowest value was found at FUI 5%, which is quite similar to the results of Oh et al. [13], where the author reported a higher spread factor in the control than in the treatments. This can be done by entrapping seaweed powder or fragments with other ingredients, and pulling them together into a coherent cookie mass; therefore, the spreading factor of the seaweed-supplemented groups was also found to be significantly lower than that of the control group.

The textural properties of cookie dough were further characterized by rheological tests of adhesiveness (the property of sticking together), cohesiveness (energy required for breaking down the internal structure), springiness (ability to spring back after first compression) and gumminess (energy required for breaking down semi-solid food for swallowing), as reported previously on semi-solid food items [31]. Comparing with control cookies, a significant increase of adhesiveness and gumminess were observed in PUI 2.5% groups but not FUI 5%. Moreover, cohesiveness was significantly reduced in cookie dough prepared from PUI and FUI, as compared with the control. These changes in UI-added cookies may be due to the presence of various viscous materials present in seaweed [2]. However, the springiness of dough was significantly decreased in FUI 5% group, as because, it contained fragmented seaweed that disintegrates the dough materials in our study.

### 3.3. Textural Characteristics of Cookies Prepared with PUI and FUI

**Figure 2:**
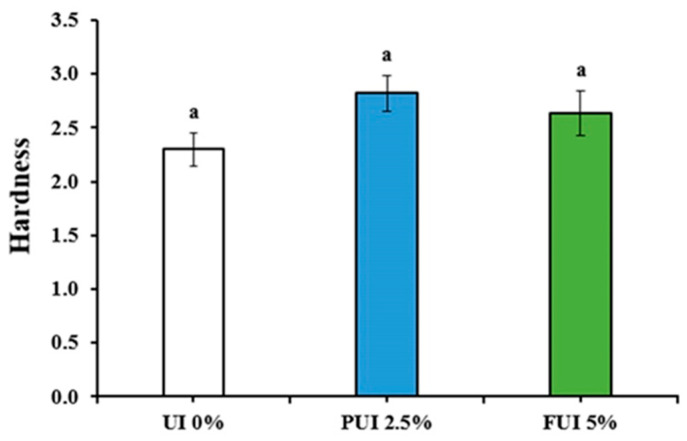
Hardness (kg/cm^2^) comparison of cookies prepared with UI 0%, PUI 2.5%, and FUI 5% UI. Data represent the mean ± SE of 3 observations, where groups not sharing a letter are expressed as significantly different (*p* < 0.05).

The texture properties of a food product are one of the most important physical quality attributes, by which, a consumer can easily evaluate [32]. Hardness is the textural property that is attracting more attention when evaluating baked goods as it is closely related to human perception of freshness [33]. Figure 2 shows the hardness of cookies made from UI 0%, PUI 2.5% and FUI 5% UI and the values were taken as 2.30, 2.82 and 2. 64 kg/cm² recorded. Although the values were not significantly different between treatments. This result agreed with the results of Mancebo et al. [34] where the author reported increased biscuit hardness from the addition of both insoluble and soluble fiber. This could be due to the changes in moisture content and network structure in these cookies caused by gums and polysaccharides contained in UI seaweed. In the case of PUI 2.5%, it was reported that higher hardness could be due to the higher solubility of powdered seaweed, whereas seaweed fragments have lower binding capacity and solubility.

### 3.4. Color Characteristics of Cookies Prepared with PUI and FUI

**Figure 3:**
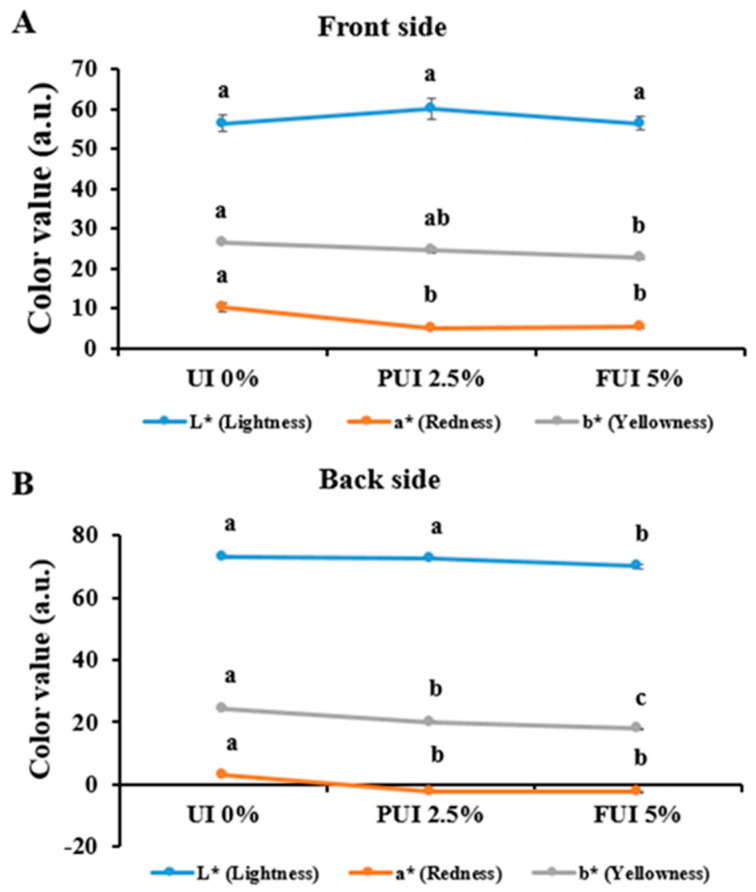
Changes in color values in both (**A**) front side and (**B**) back side of cookies prepared with UI 0%, PUI 2.5%, and FUI 5% UI. The dimension L* means lightness, with 100 for white and 0 for black appearance; a* indicates redness for positive value and greenness for negative value, b* indicates yellowness for positive value and blueness for a negative value. Data represent the mean ± SE of 3 observations, where groups not sharing a letter are expressed as significantly different (*p* < 0.05).

Seaweeds generally obtain chlorophyll or carotenoid pigments, providing them a unique green, brown, or red color, which are discolored during cooking and storage [35]. On the front side of the cookies, the L* (Lightness) value of PUI 2.5% was considerably higher compared to PUI 0% and FUI 5% but not statistically significant (Figure 3A). On the back side, L * (Lightness) was found higher in the control group and the significantly lowest value was recorded in FUI 5% (Figure 3B). This is indicative of the existence of a browner color on top of the cookie due to the cooking process and the contact with the hot air, since it has a higher temperature than the oven tray. The current finding was quite similar to those reported by Pereira et al. [36] and Adeola and Ohizua [37], where the authors reported lower L * (Lightness) in the top side of cookies when compared to the back sides. The a * (Redness) and b * (Yellowness) colors of cookies on both front and back sides were statistically significant in control (0% UI) cookies than that of seaweed cookies of PUI 2.5% and FUI 5%, respectively. This result is more or less similar to the observations made by Pereira et al. [36] but in contrast to those of Adeola and Ohizua [37]. This can be due to the difference in heat transfer, temperature, moisture content, and air velocity.

### 3.5. Nutritional Characteristics of Cookies Prepared with PUI and FUI

**Figure 4:**
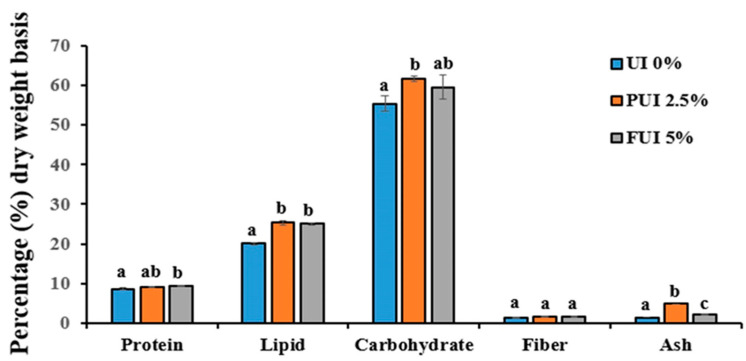
Nutritional composition of cookies prepared with UI 0%, PUI 2.5%, and FUI 5% UI. Data represent the mean ± SE of 3 observations, where groups not sharing a letter are expressed as significantly different (*p* < 0.05).

The nutritional value, particularly protein, lipid and ash, was significantly increased in cookies with PUI 2.5% and FUI 5% compared to control cookies (Figure 4). This study is similar with the study of Mamat et al. [38] as they found inclusion of seaweed improves the proximate composition of bakery product muffin. The protein content of cookies increased with the increase of UI concentrations. This result is similar to the findings of Kabirullah et al. [27] and Vijay [14], where the authors reported the protein percentage as 6.88–11.78% and 7.34–10.20%, respectively. Lipid content was found at 25.39% and 25.04% in PUI 2.5% and FUI 5% treated groups, respectively, and showed significantly higher (*p* < 0.05) compared to the control. This was similar to the findings of Kabirullah et al. [27] and Vijay [14], where the authors reported the lipid percentage as 23.02%, 5.66–26.67%, and 26.30–27.96%, respectively. An increase in lipid content might be an indication of the presence of PUFA [39]. Ash content varied from 1.45 to 4.81, where the highest value was noted in PUI 2.5% and found significantly higher (*p* < 0.05) compared to FUI 5% and control, respectively, indicating the incorporation of two different forms of seaweeds to cookies affected the ash content that might be the cause of differential particle sizes. Due to increased particle size of fragmented seaweed, water-holding and swelling capacities are high. In support, Jongaroontaprangsee et al. [40] reported similar findings where the outer leaves of cabbage were found to be lower in water-holding and swelling properties with decreasing particle size. In recent study, *U. intestinalis*, formerly known as *Enteromorpha intestinalis* from Bangladesh showed a considerable amount of mineral elements in dry matter basis (DM), among them, Ca (190.45 mg% DM), Fe (98.27 mg% DM), Cu (1.92 mg% DM), Zn (1.66 mg% DM), Na (12.33 mg% DM) and K (238.05 mg% DM) were the most dominant ones [9]. The study found higher minerals than the finding of Vijay [14], where the authors reported the ash percentage as 0.40–1.92%, respectively. These findings suggested that cookies from 2.5% PUI and 5% FUI have greater potential in the substantial level of essential minerals, thereby attracting consumer interest as UI cookies are more nutritious in comparison to conventional cookies. More precisely, it can be said that cookies made of 2.5% PUI was the best of the other inclusion level and resulted in more than double the ash content and almost similar lipid enhancement compared to 5% FUI cookies.

### 3.6. Freshness Characteristics of Cookies Prepared with PUI and FUI

**Figure 5:**
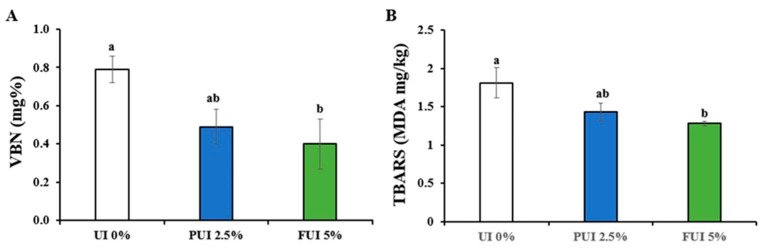
Changes in (**A**) VBN and (**B**) TBARS values in cookies prepared with UI 0%, PUI 2.5%, and FUI 5% UI. Data represent the mean ± SE of 3 observations, where groups not sharing a letter are expressed as significantly different (*p* < 0.05).

Increased VBN value occurs when proteins are broken down by microorganisms or endogenous enzymes during processing and storage, which is an important indicator for freshness of food products. Moreover, cookies contain high levels of fat, leading to increased sensory attributes, but they are sources of unwanted compounds, such as lipid oxidation products, which can be monitored by TBARS values [23]. The VBN and TBARS values of fishery products for acceptance limit for human consumption have been reported as 30–35 mgN/100 g and 10–20 mg malonaldehyde/kg, respectively [19,21,41]. Both normal cookies and fortified with UI showed a very good and acceptable limit of chemical attributes in the contents of VBN (Figure 5A) and TBARS (Figure 5B). Moreover, only cookies in the 5% FUI group had a significant (*p* < 0.05) variation in reducing the VBN (0.40 ± 0.13) and TBARS (1.28 ± 0.03) values, compared with normal cookies. These results were in accordance with the previous study of Abraha et al. [41], who found in their cookies fortified with sturgeon fish fillet powder had acceptable VBN (0.47–1.09 mgN/100 g) and TBARS (0.13–1.27 mg malonaldehyde/kg) values. However, in a recent study conducted by Oh et al. [13] prepared cookies with various seaweeds from the Korean coast, VBN and TBARS values were not assessed during quality characterization. The results of the physicochemical characteristics of cookies indicated that UI addition may help to keep the freshness over longer periods of time by suppressing the various chemical reactions, possibly through the antioxidative and anti-microbial actions [42]. VBN contributes to the generation of ammonia, biogenic amines and other products of amino acid deamination and decarboxylation and it has a positive association with the activity of proteolytic enzymes present in food products [43]. Therefore, the addition of UI to cookies resulting in the decreased VBN values might be the cause of suppressing the proteolytic enzymes in cookies. It is well known that seaweeds possess a rich source of natural and bioactive antioxidants such as sulphated polysaccharides, phenolics and flavonoids, carotenoid pigments, dietary fiber etc. [9,12]. These antioxidant substances in seaweed would play a role in chelating free ions released from heat processing and, thus, inhibit degradation products of fats, collectively known as TBARS, when added to cookies.

### 3.7. Fatty Acid Profiles of UI Seaweed

**Figure 6:**
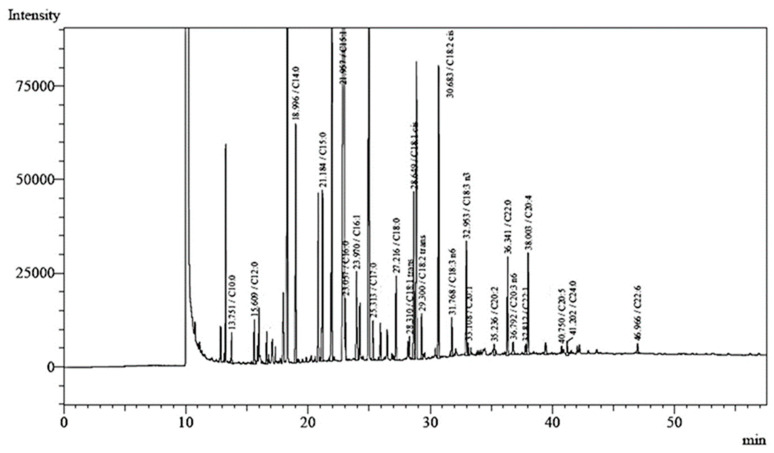
Gas chromatogram of injected FAMEs from UI with major fatty acids peaks.

Since the earlier study of nutritional analysis showed high lipid contents of UI-treated cookies, here, we further evaluated the full fatty acid profile of UI to give potential insights into the health-beneficial effects after inclusion in cookies. Moreover, the present study reported for the first time, so far, on fatty acid profile of UI from Bangladesh coast. The fatty acid contents of UI were shown in Table 3 and the gas chromatographic profile of UI was also presented in Figure 6. A total of 24 fatty acids were identified in the oil-extracted sample of UI. The most abundant fatty acids were detected as pentadecenoic acid (286.86 ± 1.01 μg/g DW), followed by linoleic acid (224.52 ± 0.93 μg/g DW), myristic acid (165.72 ± 0.59 μg/g DW), pentadecylic acid (140.93 ± 0.30 μg/g DW), and so on. The amount of total saturated, mono-unsaturated, omega-3 fatty acids and omega-6 fatty acids were reported to be 641.84 (36.14%), 563.70 (31.74%), 133.81 (7.54%) and 436.30 (24.57%) μg/g DW, respectively. Seaweed is a significant source of polyunsaturated fatty acids [44]. The total polyunsaturated fatty acids were found to be 570 μg/g DW, which correspondent to 32.11%. Among individual polyunsaturated fatty acids identified in UI, linoleic acid (12.64%) was the most abundant one, followed by α-Linolenic acid (5.29%). The results suggested that the amount of total fatty acids in UI contributed the majority to the total lipid content analyzed previously. It is the first report on the fatty acids profiles of UI from Bangladesh. Among the green seaweed tested by Cardoso et al. [45], omega-3 and-6 fatty acids were found in *U. intestinalis* (14.6 and 10.7, respectively), *U. prolifera* (14.0 and 24.7 %, respectively) and *U. lactuca* (14.1 and 12.1, respectively); our findings were greatly supported by this study. Although, the amount of fatty acids in dry weight mass is absent. Moreover, another study conducted on green seaweed, *U. lactuca* was in agreement with our findings [46]. Since polyunsaturated fatty acids in UI possessed a substantial level offering a wide spectrum of health effects such as cardiovascular disease mitigation [47], neuroprotective [48] and neurotrophic [49] effects, and obesity [47] and diabetes [50] management, upon consumption, especially in the form of inclusion to dietary cookies in an approach of the present study.

## 4. Conclusions

The study was conducted to develop value-added health-functional seaweed-based cookies. The fishy smell was significantly reduced in cookies with 2.5% PUI and 5% FUI. It was found that the addition of 2.5% PUI and 5% FUI to the cookies was similar to the control cookies in terms of sensory properties. In addition, a significantly higher nutrient composition (protein, lipid and ash) was observed in seaweed-based cookies made from both PUI and FUI. At 2.5% PUI and 5% FUI, cookies had significant and acceptable physicochemical and structural properties. Furthermore, the addition of UI to cookies resulted in a high lipid content, the fatty acid profile of which was further analyzed, which was the first time for UI from Bangladesh coastline. UI has a significant content of polyunsaturated fatty acids, either in dry matter or as a percentage of total fatty acids, confirming its biofunctional properties when added to cookies. However, whether thermal processing could preserve the beneficial fatty acids in cookies and how long UI could be effective in preserving biscuit quality during storage should warrant for further study. These results suggest the possibility of developing baked goods with highly nutritious and healthy functional properties made from *Ulva intestinalis* available on the Bangladesh coast.

## Figures and Tables

**Table 1:** Ingredients of UI seaweed-based cookie.

Ingredients	Brand Name	Amount
Wheat flour	Teer	90~100%
Seaweed	Collected from Coastal Site	0~10%
Baking powder	Foster Clark’s Baking Powder	0.5 g
Baking soda	Pure Baking Soda	0.05 g
Icing sugar	Fresh Refined Sugar	15 g
Salt	ACI PURE Salt	1 g
Milk powder	Diploma Instant Full Cream Milk Powder	8 g
Flavor	Foster Clark’s Lemon Flavor	6 mL
Lemon juice	Extracted from locally available Lemon	2.5 mL
Butter	Aarong Dairy Butter	10 g
Oil	Rupchanda Fortified Soyabean Oil	25 mL

**Table 2:** Quality characteristics of value-added cookies with UI seaweed.

Physical Properties	UI 0%	PUI 2.5%	FUI 5%
Dough pH	6.59 ± 0.06 ^a^	6.53 ± 0.05 ^b^	6.51 ± 0.08 ^b^
Dough density (g/cm^3^)	1.33 ± 0.03 ^a^	1.32 ± 0.02 ^a^	1.33 ± 0.03 ^a^
Cookie density (g/cm^3^)	0.98 ± 0.03 ^a^	0.94 ± 0.02 ^a^	0.91 ± 0.05 ^a^
Cookie moisture (%)	3.57 ± 0.03 ^a^	3.35 ± 0.02 ^a^	3.02 ± 0.10 ^b^
Baking loss (%)	8.22 ± 0.52 ^a^	7.84 ± 0.23 ^a^	8.10 ± 0.28 ^a^
Height (mm)	6.6 ± 0.24 ^a^	6.6 ± 0.24 ^a^	6.8 ± 0.20 ^a^
Diameter (mm)	51.2 ± 0.83 ^a^	51 ± 0.10 ^a^	51.2 ± 0.83 ^a^
Spread factor	7.81 ± 0.35 ^a^	7.71 ± 0.33 ^a^	7.56 ± 0.27 ^a^
Adhesiveness (mJ)	0.42 ± 0.05 ^a^	0.87 ± 0.15 ^b^	0.72 ± 0.11 ^bc^
Cohesiveness	0.45 ± 0.02 ^a^	0.26 ± 0.02 ^b^	0.16 ± 0.01 ^c^
Springiness (mm)	3.05 ± 0.29 ^a^	3 ± 0.49 ^a^	1.75 ± 0.08 ^b^
Gumminess (g)	360.89 ± 7.59 ^a^	712.56 ± 75.52 ^b^	457 ± 26.19 ^a^

Data represent the mean ± SE of 3 observations, where groups not sharing a letter are expressed as significantly different (*p* < 0.05). UI: *Ulva intestinalis*, PUI: powdered *Ulva intestinalis*, FUI: fragmented *Ulva intestinalis.*

**Table 3:** Fatty acid contents of UI dried powder.

Systematic Name	Common Name	Abbreviation	Amount (μg/g DW)
Decanoic acid	Caproic acid	C10:0	34.05 ± 0.23
Dodecanoic acid	Lauric acid	C12:0	45.27 ± 0.14
Tetradecanoic acid	Myristic acid	C14:0	165.72 ± 0.59
Pentadecylic acid		C15:0	140.93 ± 0.30
Hexadecanoic acid	Palmitic acid	C16:0	41.20 ± 0.46
Heptadecanoic acid	Margaric acid	C17:0	36.76 ± 0.12
Octadecanoic acid	Stearic acid	C18:0	76.04 ± 0.29
Docosanoic acid	Behenic acid	C22:0	91.49 ± 0.75
Tetracosanoic acid	Lignoceric acid	C24:0	10.38 ± 0.61
∑ **SAFA**			**641.84**
Pentadecenoic acid		C15:1	286.86 ± 1.01
Hexadecenoic acid	Palmitoleic acid	C16:1	83.96 ± 0.29
Cis-9-Octadecenoic acid	Oleic acid	C18:1 cis	140.14 ± 0.55
Trans-9-Octadecenoic acid	Elaidic acid	C18:1 trans	23.32 ± 0.10
Cis-11-Eicosenoic acid		C20:1	13.86 ± 0.25
Cis-13-Docosenoic acid	Erucic acid	C22:1	15.56 ± 0.63
∑ **MUFA**			**563.70**
All cis-9,12,15-Octadecatrienoic acid	α -Linolenic acid	C18:3	94.06 ± 0.24
All cis-5,8,11,14,17-Eicosapentenoic acid	EPA	C20:5	24.42 ± 1.13
All cis-4,7,10,13,16,19-Docosahexenoic acid	DHA	C22:6	15.33 ± 0.23
∑ **PUFA (Omega-3)**			**133.81**
All cis-9,12-Octadecadienoic acid	Linoleic acid	C18:2 cis	224.52 ± 0.93
All trans-9,12-Octadecadienoic acid	Linolelaidic acid	C18:2 trans	39.17 ± 0.21
All cis-6,9,12-Octadecatrienoic acid	γ-Linolenic acid	C18:3	37.08 ± 0.03
All cis-11,14-Eicosadienoic acid		C20:2	17.55 ± 0.03
All cis-8,11,14-Eicosatrienoic acid	Dihomogammalinolenic acid	C20:3	22.73 ± 1.10
All cis-5,8,11,14-Eicosatetraenic acid	Arachidonic acid	C20:4	95.26 ± 0.70
∑ **PUFA (Omega-6)**			**436.30**

SAFA: saturated fatty acids, MUFA: mono-unsaturated fatty acids, PUFA: poly-unsaturated fatty acids.

## Data Availability

Data supporting the reported results are available upon request.
